# Molecular evolution and expression profile of the chemerine encoding gene RARRES2 in baboon and chimpanzee

**DOI:** 10.1186/s40659-015-0020-0

**Published:** 2015-06-12

**Authors:** Rafael González-Alvarez, María de Lourdes Garza-Rodríguez, Iván Delgado-Enciso, Víctor Manuel Treviño-Alvarado, Ricardo Canales-Del-Castillo, Laura Elia Martínez-De-Villarreal, Ángel Lugo-Trampe, María Elizabeth Tejero, Natalia E. Schlabritz-Loutsevitch, María Del Refugio Rocha-Pizaña, Shelley A. Cole, Diana Reséndez-Pérez, Mario Moises-Alvarez, Anthony G. Comuzzie, Hugo Alberto Barrera-Saldaña, Raquel Garza-Guajardo, Oralia Barboza-Quintana, Irám Pablo Rodríguez-Sánchez

**Affiliations:** Universidad Autónoma de Guadalajara, Facultad de Medicina, Avenida Patria 1201, Colonia Lomas del Valle, CP 45129 Zapopan, Jalisco México; Universidad Autónoma de Nuevo León, Facultad de Medicina, Departamento de Bioquímica y Medicina Molecular, Av. Madero y Gonzalitos s/n, Colonia Mitras Centro, CP 64460 Monterrey, Jalisco México; Universidad de Colima, Facultad de Medicina, Av. Universidad 333, Colonia Las Víboras, CP 38040 Colima, Colima México; Tecnológico de Monterrey campus Monterrey, Av. Eugenio Garza Sada 2501, Colonia Tecnológico, CP 64849 Monterrey, Nuevo León México; Universidad Autónoma de Nuevo León, Facultad de Ciencias Biológicas, Av. Pedro de Alba, Ciudad Universitaria, CP 66450 San Nicolás de los Garza, Nuevo León México; Universidad Autónoma de Nuevo León, Hospital Universitario Dr. José Eleuterio González, Departamento de Genética, Av. Madero y Gonzalitos s/n, Colonia Mitras Centro, CP 64460 Monterrey, Nuevo León México; Centro Mesoamericano de Estudios en Salud Pública y Desastres (CEMESAD, Nodo Tapachula), Universidad Autónoma de Chiapas, CP 30798 Tapachula, Chiapas México; Instituto Nacional de Medicina Genómica. Periférico Sur 4809, Colonia Arenal Tepepan, CP 14610 Delegación Tlalpan, Distrito Federal México; Texas Tech University Health Sciences Center at the PB, Odessa, TX USA; Auxology and Metabolism Working Group, Department of Genetics, Southwest Foundation for Biomedical Research, San Antonio, TX USA; Universidad Autónoma de Nuevo León, Facultad de Medicina, Departamento de Anatomía Patológica y Citopatología, Av. Madero y Gonzalitos s/n, Colonia Mitras Centro, CP 64460 Monterrey, Jalisco México

**Keywords:** Retinoic acid receptor, Responder protein, Primate, Chemerin, TIG2, Tazarotene-induced gene 2 protein, Gene expression

## Abstract

**Background:**

Chemerin, encoded by the retinoic acid receptor responder 2 (RARRES2) gene is an adipocytesecreted protein with autocrine/paracrine functions in adipose tissue, metabolism and inflammation with a recently described function in vascular tone regulation, liver, steatosis, etc. This molecule is believed to represent a critical endocrine signal linking obesity to diabetes. There are no data available regarding evolution of RARRES2 in non-human primates and great apes. Expression profile and orthology in RARRES2 genes are unknown aspects in the biology of this multigene family in primates. Thus; we attempt to describe expression profile and phylogenetic relationship as complementary knowledge in the function of this gene in primates. To do that, we performed A RT-PCR from different tissues obtained during necropsies. Also we tested the hypotheses of positive evolution, purifying selection, and neutrality. And finally a phylogenetic analysis was made between primates RARRES2 protein.

**Results:**

RARRES2 transcripts were present in liver, lung, adipose tissue, ovary, pancreas, heart, hypothalamus and pituitary tissues. Expression in kidney and leukocytes were not detectable in either species. It was determined that the studied genes are orthologous.

**Conclusions:**

RARRES2 evolution fits the hypothesis of purifying selection. Expression profiles of the RARRES2 gene are similar in baboons and chimpanzees and are also phylogenetically related.

## Background

Chemerine is a protein that initiates chemotaxis via the ChemR23 - G protein-coupled seven-trans-membrane domain receptor ligand, which has been classified as an adipokine due to its role in adipocyte differentiation and glucose uptake [1]. It also plays a potential role in controlling immune responses at sites of tissue injury and inflammation [2], including chronic inflammation of adipose tissue in obesity [1, 3–5]. Chemerine has also been suggested as an essential endocrine signal, linking obesity to insulin resistance [3, 6–8], therefore it is an independent biomarker of metabolic syndrome [9–12]. In addition to adipose tissue, chemerine plays an important role in metabolic regulation in the liver and skeletal muscle [6, 13]. Recently a novel role for chemerine as a stimulator of angiogenesis was identified [9].

The retinoic acid receptor responder protein 2 (RARRES2) gene (also named RAR-responsive protein TIG2, chemerin and tazarotene-induced gene 2 protein), which encodes chemerine, is located in chromosome 7 at 7q36.1 in humans. RARRES2 mRNA is highly expressed in white adipose tissue, liver and lungs, while the mRNA for chemerine receptor is predominantly expressed in immune cells and adipose tissue [14–20].

The study of the evolution of Old World primates (OWM) and great apes has been an excellent approach to understand human pathology such as metabolic syndrome. Currently, the baboon (*Papio spp*) has been proven to be an ideal model to study metabolism disturbances. Previous studies have found a substantial variation in weight and body composition in adult baboons sharing the same diet and living conditions. Baboons spontaneously develop obesity [21], type 2 diabetes mellitus (T2DM) [22] and a metabolic syndrome-like phenotype has been described in this species [23]. While the RARRES2 gene sequence has been described in humans, there is no information available regarding baboon and chimpanzee. The present study analyzed the expression profile and phylogenetic relationship of the RARRES2 gene from baboon and chimpanzee.

## Results

### Expression profile

In the PCR from genomic DNA, a single band of 3,257 bp was visualized for both primates (data not shown). In a similar manner in the PCR from cDNA, only one product of 641 bp was observed for both primates. PCR product sizes and nucleotide sequence are not sufficient to determine absence of paralogous genes, but with the evidence given at the moment could be that there is only one RARRES2 gene for each of the two species studied, baboon and chimpanzee. Both genes are organized in six exons and five introns of variable size: exon 1,110 pb; intron A, 948 pb; exon 2, 194 pb; intron B, 231 pb; exon 3, 105 pb; intron C, 1018 pb; exon 4, 96 pb; intron D, 244 pb; exon 5, 127 pb; intron E, 175 pb; and exon 6, 121 pb.

**Fig. 1:**
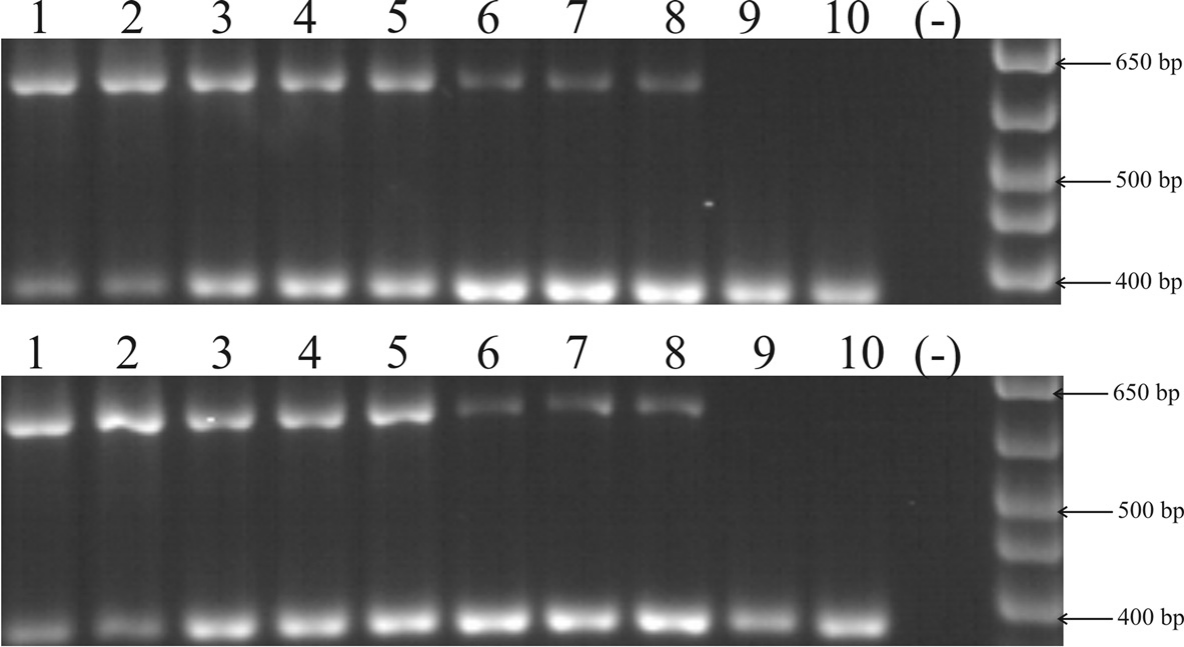
RT-PCR profile expression screening of RARRES2 gene. Top panel shows RT-PCR assays from baboon’s tissues. Button panel shows RT-PCR assays from chimpanzee’s tissues. In both panels, the lanes 1 to 10 represent: liver, lung, adipose tissue, ovary, pancreas, heart, hypothalamus, pituitary gland, kidney and leucocytes. Lanes (−) indicate the negative RT-PCR controls. Positives controls (324 bp) were co amplified with samples (641 bp)

For each species, RARRES2 transcripts were amplified from liver, lung, adipose tissue, ovary, pancreas, heart, hypothalamus, and pituitary gland. Expression in kidney and leucocytes was not detected. Negative and positive controls gave the expected results (Fig. 1). All PCR products were cloned and sequenced. For every primate, at least three independent clones were sequenced for each gene and transcript. Baboon and chimpanzee mRNA sequences resulted identical for all tissues.

### Phylogenetic analysis

**Fig. 2:**
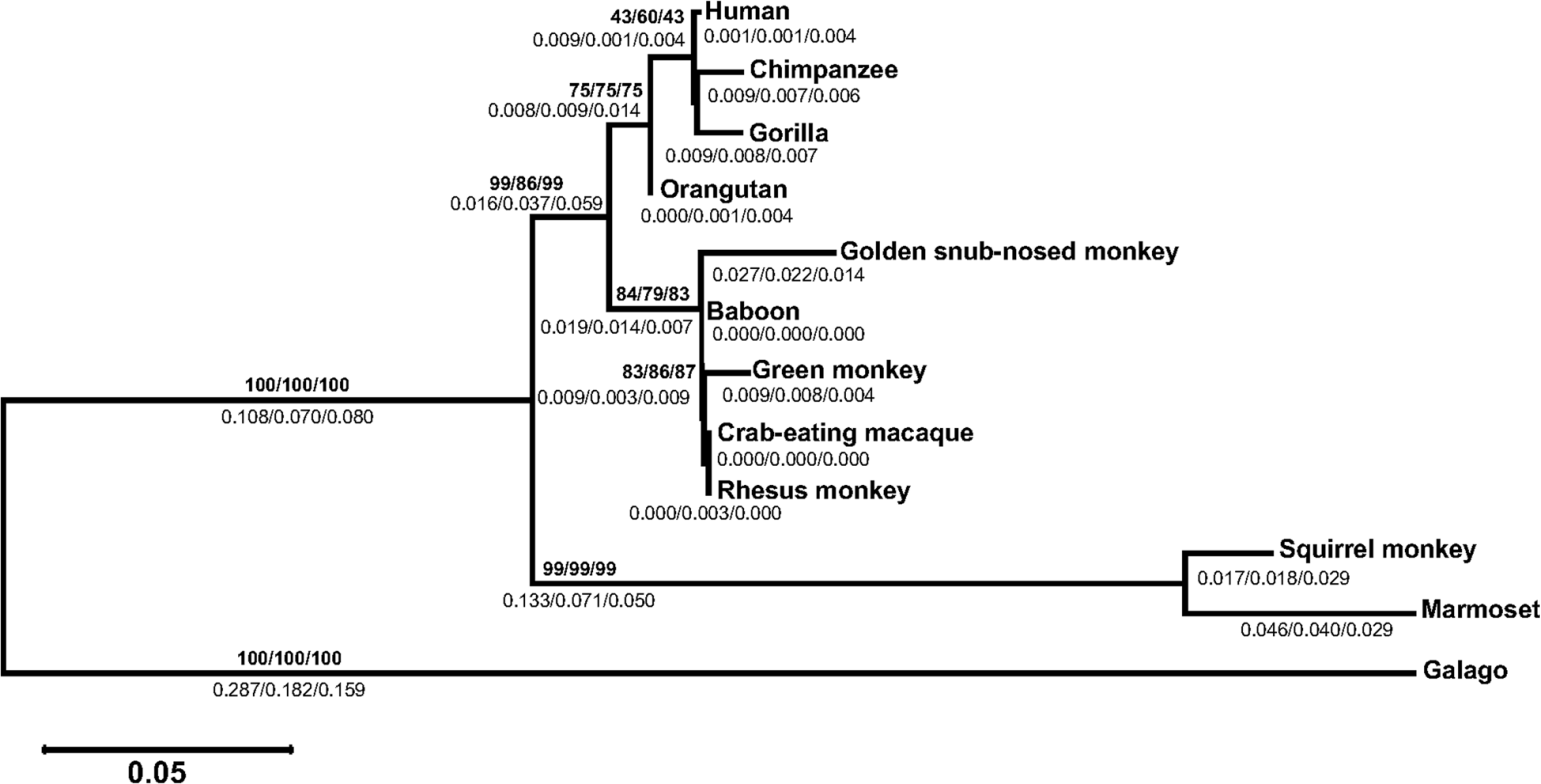
Phylogenetic tree of RARRES2 proteins from various primates. The tree was built using MEGA version 6.06 by the ML, NJ and UPGMA methods and further bootstrap analysis of 1000 replicas. Numbers in the branches indicate Bootstrap value (bold) and below the branches length, both estimated by three methods (ML/NJ/UPGMA). Clades are in linage specific manner, apes, OWM, NWM and lemur (out-group)

The phylogenetic tree (Fig. 2) shows four clades in a lineage specific manner. These clades correspond to apes, OWM, NWM and lemur (out-group). It confirms the orthology between primate RARRES2 genes. Bootstrap values are shown on the tree’s branches. Similar results were obtained using Maximum Likelihood (ML), Neighbor-Joining (NJ) and UPGMA phylogenetic methods. We confirmed that RARRES2 evolution fits the hypothesis of purifying selection (*P* < 0.05) for OWM (except for green monkey) and apes. Since for RARRES2 from NWM the tests did not yield statistically significant differences (*P* > 0.05), at this moment it is unclear which forces govern their evolution. Neutrality is observed for orangutan, rhesus monkey and crab-eating macaque RARRES2 genes (Table 1).

**Table 1:** Test to determine the evolutionary forces that underlie the RARRES2 primate genes using the Li-Wu-Luo method (Kimura 2-parameters)

Specie			Positive	*P* value for Purifying d_N_	Neutrality
	d_N_	d_S_	d_N_ > d_S_	< d_S_	d_N_ = d_S_
*Homo sapiens*	0.1930	0.3637	1.0000	**0.0277**	0.0554
*Pan troglodytes*	0.1884	0.3618	1.0000	**0.0256**	0.0511
*Gorilla gorilla*	0.1897	0.3566	1.0000	**0.0293**	0.0587
*Pongo abelii*	0.1928	0.3851	1.0000	**0.0194**	**0.0388**
*Macaca mulatta*	0.1834	0.3671	1.0000	**0.0222**	**0.0445**
*Macaca fascicularis*			1.0000	**0.0222**	**0.0445**
*Rhinopithecus roxellana*	0.1834	0.3671	1.0000	**0.0385**	0.0770
*Chlorocebus sabaeus*	0.1892	0.3407	1.0000	0.0521	0.1042
	0.1889	0.3223			
*Papio anubis*	0.1834	0.3426	1.0000	**0.0314**	0.0628
*Saimiri boliviensis*	0.2250	0.3445	1.0000	0.0901	0.1801
*Callithrix jacchus*	0.2385	0.3834	1.0000	0.0685	0.1369

We tested whether d_N_ is significantly greater, lower or equal, respectively, than d_S_ using codon-based Z test of selection as implemented in the MEGA 6.06 software. Differences were considered statistically significant at a *P* <0.05

Bold numbers indicate *P* < 0.05

## Discussions

### mRNA tissue expressions

This is the first report showing the expression profile of the novel RARRES2 mRNA in different tissues from baboon and chimpanzee. The novel finding of this study is that in baboon and chimpanzee, there is expression in the pituitary gland, while in humans RARRES2 was not detected in this tissue. In humans, expression in kidney has been reported (ETS profile at the NCBI, UniGene), while in baboon and chimpanzee in our study it was not detected. To address this difference studies such as; promoters alignment, bioinformatics analyses to predict transcptional factors bindings sites, searching CpG islands susceptible to be methylated, as others; are needed.

Similarity in the expression pattern suggests that mechanisms of regulation of gene expression are similar in the three species. It also suggests that the RARRES2 gene might have similar physiological effects. However more studies in functional differences are needed, such as studying the expression profile under different conditions such as insulin resistance, obesity, fasting or others associated with metabolic syndrome.

### Gene structure

We found that the RARRES2 cDNA sequence in baboon and chimpanzee have a high similarity to others apes. The conservation of exon–intron boundaries and absence of obvious mutations suggest that the baboon and chimpanzee RARRES2 genes are functional.

### Phylogenetic analysis

Positive selection (d_N_ > d_S_) implies that the substitutions mostly non-synonymous are functional and benefit the organism, conferring some evolutionary advantage. While purifying selection (d_N_ < d_S_) indicates that evolutionary pressure has been relaxed. The d_N_ and d_S_ rates show that the evolutionary force actually acting on these is the purification of the selection (*P* < 0.05). Fit hypothesis of purifying of selection is a clue that these genes are functional in the studied species, because there are not functional genes that do not fit this hypothesis. Although we did not do an analysis between the expression profiles of primate RARRES2 genes and their putative binding sites for known transcriptional factors, we speculate that this evolutionary force has been relaxed perhaps because in apes and OWM the functions and/or regulatory mechanisms are not necessarily the same as those governing NWM or lemurs. However, more research is needed to investigate the role of this molecule in physiology and regulatory expression profiles.

The phylogenetic relationship between NWM, OWM and apes RARRRES proteins was determined to evaluate their evolution in primates. The phylogenetic tree (Fig. 2) showed three clades in a linage-specific manner. These clades corresponded to NWM, OWM and apes, finally galago (out-group). Tree’s topology, length’s branches and Bootstrap values are similar using either phylogenetic method (ML/NJ/UPGMA). This confirms orthology within the RARRES gene. Bootstrap values are shown bold on the branches of the tree; below of Bootstrap values are shown branches length estimated by the same methods (ML/NJ/UPGMA).

## Conclusions

We found that there is only one RARRES2 gene for baboon and chimpanzee. RARRES2 transcripts were present in liver, lung, adipose tissue, ovary, pancreas, heart, hypothalamus and pituitary tissues from both primates. The d_N_ and d_S_ rates conclusively show that these genes have no positive selection. RARRES2 evolution fits the hypothesis of purifying selection. The studied sequences are clearly orthologous.

## Methods

### Animal housing

The baboons (*Papio anubis*) and chimpanzees (*Pan troglodytes*) were housed at the Southwest National Primate Research Center in San Antonio, TX; an AAALAC-accredited facility at the Texas Biomedical Research Institute. These animals are housed with indoor-outdoor access in standard stainless steel cages with covered shelters equipped with external containers for food and *ad libitum* access to water. The protocol was approved by the IACUC of the Texas Biomedical Research Institute for all procedures.

### Tissue harvesting and processing

The liver, lung, adipose tissue, ovary, pancreas, heart, hypothalamus, pituitary gland, kidney and total leukocytes were collected at routine necropsy and stored at −80 °C until further evaluation. Baboon tissues came from three different animals, while chimpanzee tissues came from a single female that underwent humane euthanasia due to a complication of diabetes. Total RNA and DNA of the different tissues and leukocytes were extracted with Trizol reagent according to the manufacturer’s instructions (Invitrogen, Carlsbad, CA). RNA was treated with RQ1 DNase (Promega, Madison, WI) for 15 min at 37 °C to remove traces of genomic DNA. The purity and integrity of RNA and DNA were assessed using standard spectrophotometry methods with NanoDrop equipment (Thermo Scientific, Wilmington, DE) and agarose gel electrophoresis.

### Reverse transcription (RT), polymerase chain reaction (PCR), molecular cloning and nucleotide sequencing

Total RNA from frozen tissues (~1.0 μg) was retro-transcribed according to the manufacturer’s instructions with a High Capacity cDNA Reverse Transcription (RT) kit (Applied Biosystems, Foster City, CA) into a 50 μL final volume. A primer set to amplify baboon and chimpanzee’s RARRES2 transcripts was designed using RARRES2 sequences from rhesus monkey (*Macaca mulatta*) and humans (*Homo sapiens*) as templates. Sequence templates are deposited in the NCBI (Table 2) [http://www.ncbi.nlm.nih.gov/]. Such design used an online tool [24]. Sense primer (5′-GGACAGCGAGGCCAAGAT-3′) hybridizes in exon one at nucleotide 68 prior to the start codon (AUG). Antisense primer (5′-CTGGGGTCTTCCACTGGTTAC-3′) hybridizes in exon six at nucleotide after the stop triplet (UAA) (positions referent to the RNA molecule). For PCR we used 5 μL of RT reaction or 200 ng of genomic DNA as templates, 10 μM of each primer and the 2X PCR master mix kit (Qiagen, Valencia, CA). The reactions were performed in a 25 μL final volume carried out in a Veriti thermal cycler (Applied Biosystems, Foster City, CA), with the following amplification programs: first for RT samples, an initial denaturalization step for 3 min at 94 °C; then 30 cycles of 30 s at 94 °C, 30 s at 58 °C, and 30 s at 72 °C; finally an elongation step of 6 min at 72 °C. Second for genomic DNA samples, an initial denaturalization step for 4 min at 94 °C; then 30 cycles of 1 min at 94 °C, 30 s at 58 °C, 2.5 min at 72 °C; finally an elongation step of 15 min at 72 °C. Primers for ribosomal RNA fraction 18S (Applied Biosystems, Foster City, CA), were used as RT positive controls (324 bp in size). The amplification reactions were confirmed in 1 % agarose electrophoresis gel stained with ethidium bromide and visualized with UV light. PCR products were cloned using a 3.5 kb-XL-TOPO-vector kit and transformed in the electrocompetent bacterium *E. coli* strain Top 10. These procedures were performed according to the manufacturer’s specifications (Invitrogen, Carlsbad, CA). The positive clones were sequenced using a Big Dye terminator kit and either specific or universal (M13) primers. The reactions were analyzed with an ABI PRISM 3100 Genetic Analyzer and in its software (Applied Biosystems, Foster City, CA).

**Table 2:** Primate RARRES2 sequences from NCBI GenBank used in this study

	Accession no.		
Species	Gene	mRNA	Protein
Apes			
Human (*Homo sapiens*)	NC_000007.13	BC000069	AAH00069
Chimpanzee (*Pan troglodytes*)	NC_006474.3	XM_001134678	XP_001134678
Gorilla (*Gorilla gorilla*)	NC_018431.1	XM_004046459	XP_004046507
Orangutan (*Pongo abelii*)	NC_012598.1	NM_001134159	NP_001127631
Old World Monkeys (OWM)			
Rhesus monkey (*Macaca mulatta*)	NW_001116299.1	NM_001266683	NP_001253612
Crab-eating macaque (*Macaca fascicularis*)	NW_005093937	XM_005595678	XP_005595735
Golden snub-nosed monkey (*Rhinopithecus roxellana*)	NW_010823286.1	XM_010379730	XP_010378032
Green monkey (*Chlorocebus sabaeus*)	NC_023662.1	XM_007983416	XP_007981607
Baboon (*Papio anubis*)	NC_018154.1	XM_003896833	XP_003896882
New World Monkey (NWM)			
Squirrel monkey (*Saimiri boliviensis*)	NW_003943639.1	XM_003929843	XP_003929892
Marmoset (*Callithrix jacchus*)	NC_013903.1	XM_002751892	XP_002751938
Lemur (out group)			
Galago (*Otolemur garnettii*)	NW_003852448.1	XM_003792092	XP_003792140

### Phylogenetic analysis

The information obtained from the sequencing assays was subjected to a BLAST test to determine identity. The structures of intron-exon boundaries of each one of the baboon and chimpanzee RARRES2 genes were determined by directly retrieving information given at the NCBI server. Alignments were performed using the CLUSTAL W program [25]. GenBank accession numbers of the sequences used in this study are provided in Table 2. From amino acidic sequence, a phylogenetic tree was built with MEGA 6.06 software [26] using the Maximum Likelihood (ML), Neighbor-Joining (NJ) and UPGMA methods, thena bootstrap test was done with 1,000 replicates [27].

Seeking to identify the evolutionary forces that underlie the process of divergence in the RARRES2 primate genes, we tested the hypothesis of positive or adaptive evolution (d_N_ > d_S_), purifying selection (d_N_ < d_S_) and neutrality (d_N_ = d_S_). For this purpose; first, we calculated the non-synonymous (causes an amino acid change) and synonymous (does not cause an amino acid change) d_N_, d_S_ distances, respectively, by the the Li-Wu-Luo method (Kimura 2-parameters) [28] from RARRES2’ coding sequences from apes, OWM and NWM with their lemur’s counterpart. Second, we tested whether d_N_ is significantly greater, lower or equal, respectively, than d_S_ using codon-based Z test of selection as implemented in MEGA 6.06 software [26]. Differences were considered statistically significant at a *P* <0.05.

## Abbreviations

RARRES2: Retinoic acid receptor responder protein 2
OWM: Old World primates
T2DM: Type 2 diabetes mellitus

## References

[1] Goralski KB, McCarthy TC, Hanniman EA, Zabel BA, Butcher EC, Parlee SD, Muruganandan S, Sinal CJ (2007). Chemerin, a novel adipokine that regulates adipogenesis and adipocyte metabolism. J BiolChem.

[2] Allen SJ, Zabel BA, Kirkpatrick J, Butcher EC, Nietlispach D, Handel TM (2007). NMR assignment of human chemerin, a novel chemoattractant. Biomol NMR Assign.

[3] Ernst MC, Sinal CJ (2010). Chemerin: at the crossroads of inflammation and obesity. Trends EndocrinolMetab.

[4] Hart R, Greaves DR (2010). Chemerin contributes to inflammation by promoting macrophage adhesion to VCAM-1 and fibronectin through clustering of VLA-4 and VLA-5. J Immunol.

[5] Parolini S, Santoro A, Marcenaro E, Luini W, Massardi L, Facchetti F (2007). The role of chemerin in the colocalization of NK and dendritic cell subsets into inflamed tissues. Blood.

[6] Becker M, Rabe K, Lebherz C, Zugwurst J, Goke B, Parhofer KG (2010). Expression of human chemerin induces insulin resistance in the skeletal muscle but does not affect weight, lipid levels, and atherosclerosis in LDL receptor knockout mice on high-fat diet. Diabetes.

[7] Parlee SD, Ernst MC, Muruganandan S, Sinal CJ, Goralski KB (2010). Serum chemerin levels vary with time of day and are modified by obesity and tumor necrosis factor-{alpha}. Endocrinology.

[8] Pfau D, Stepan H, Kratzsch J, Verlohren M, Verlohren HJ, Drynda K (2010). Circulating levels of the adipokinechemerin in gestational diabetes mellitus. Horm Res Paediatr.

[9] Bozaoglu K, Segal D, Shields KA, Cummings N, Curran JE, Comuzzie AG (2009). Chemerin is associated with metabolic syndrome phenotypes in a Mexican-American population. J ClinEndocrinolMetab.

[10] Lehrke M, Becker A, Greif M, Stark R, Laubender RP, von Ziegler F (2009). Chemerin is associated with markers of inflammation and components of the metabolic syndrome but does not predict coronary atherosclerosis. Eur J Endocrinol.

[11] Lijnen HR (2008). Angiogenesis and obesity. Cardiovasc Res.

[12] Stejskal D, Karpisek M, Hanulova Z, Svestak M (2008). Chemerin is an independent marker of the metabolic syndrome in a Caucasian population--a pilot study. Biomed Pap Med FacUnivPalacky Olomouc Czech Repub.

[13] Ernst MC, Issa M, Goralski KB, Sinal CJ (2010). Chemerin exacerbates glucose intolerance in mouse models of obesity and diabetes. Endocrinology.

[14] Lin W, Chen YL, Jiang L, Chen JK (2011). Reduced expression of chemerin is associated with a poor prognosis and a lowed infiltration of both dendritic cells and natural killer cells in human hepatocellular carcinoma. Clin Lab.

[15] Bozaoglu K, Curran JE, Stocker CJ, Zaibi MS, Segal D, Konstantopoulos N (2010). Chemerin, a novel adipokine in the regulation of angiogenesis. J ClinEndocrinolMetab.

[16] Du XY, Leung LL (2009). Proteolytic regulatory mechanism of chemerin bioactivity. Acta BiochimBiophys Sin (Shanghai).

[17] Kaur J, Adya R, Tan BK, Chen J, Randeva HS, Kaur J (2010). Identification of chemerin receptor (ChemR23) in human endothelial cells: chemerin-induced endothelial angiogenesis. BiochemBiophys Res Commun.

[18] Knight A (2008). The beginning of the end for chimpanzee experiments?. Philos Ethics Humanit Med.

[19] Muruganandan S, Roman AA, Sinal CJ (2010). Role of chemerin/CMKLR1 signaling in adipogenesis and osteoblastogenesis of bone marrow stem cells. J Bone Miner Res.

[20] Yoshimura T, Oppenheim JJ (2008). Chemerin reveals its chimeric nature. J Exp Med.

[21] Comuzzie AG, Cole SA, Martin L, Carey KD, Mahaney MC, Blangero J (2003). The baboon as a nonhuman primate model for the study of the genetics of obesity. Obes Res.

[22] Banks WA, Altmann J, Sapolsky RM, Phillips-Conroy JE, Morley JE (2003). Serum leptin levels as a marker for a syndrome X-like condition in wild baboons. J ClinEndocrinolMetab.

[23] Cole SA, Martin LJ, Peebles KW, Leland MM, Rice K, VandeBerg JL (2003). Genetics of leptin expression in baboons. Int J ObesRelatMetabDisord.

[24] Boutros R, Stokes N, Bekaert M, Teeling EC (2009). UniPrime2: a web service providing easier Universal Primer design. Nucleic Acids Res.

[25] Thompson JD, Higgins DG, Gibson TJ (1994). CLUSTAL W: improving the sensitivity of progressive multiple sequence alignment through sequence weighting, position-specific gap penalties and weight matrix choice. Nucleic Acids Res.

[26] Tamura K, Stecher G, Peterson D, Filipski A, Kumar S (2013). MEGA6: Molecular Evolutionary Genetics Analysis version 6.0. MolBiolEvol.

[27] Saitou N, Nei M (1987). The neighbor-joining method: a new method for reconstructing phylogenetic trees. MolBiolEvol.

[28] Zhang J, Rosenberg HF, Nei M (1998). Positive Darwinian selection after gene duplication in primate ribonuclease genes. ProcNatlAcadSci U S A.

